# Preservation Analysis on Spatiotemporal Specific Co-expression Networks Suggests the Immunopathogenesis of Alzheimer’s Disease

**DOI:** 10.3389/fnagi.2021.727928

**Published:** 2021-09-03

**Authors:** Liyuan Guo, Yushan Liu, Jing Wang

**Affiliations:** ^1^CAS Key Laboratory of Mental Health, Institute of Psychology, Chinese Academy of Sciences, Beijing, China; ^2^Department of Psychology, University of Chinese Academy of Sciences, Beijing, China; ^3^College of Life Sciences, University of Chinese Academy of Sciences, Beijing, China

**Keywords:** Alzheimer’s disease, spatiotemporal specific coexpression networks, network preservation analysis, immune response-related pathways, non-neuron cells, key genes

## Abstract

The occurrence and development of Alzheimer’s disease (AD) is a continuous clinical and pathophysiological process, molecular biological, and brain functional change often appear before clinical symptoms, but the detailed underlying mechanism is still unclear. The expression profiling of postmortem brain tissue from AD patients and controls provides evidence about AD etiopathogenesis. In the current study, we used published AD expression profiling data to construct spatiotemporal specific coexpression networks in AD and analyzed the network preservation features of each brain region in different disease stages to identify the most dramatically changed coexpression modules and obtained AD-related biological pathways, brain regions and circuits, cell types and key genes based on these modules. As result, we constructed 57 spatiotemporal specific networks (19 brain regions by three disease stages) in AD and observed universal expression changes in all 19 brain regions. The eight most dramatically changed coexpression modules were identified in seven brain regions. Genes in these modules are mostly involved in immune response-related pathways and non-neuron cells, and this supports the immune pathology of AD and suggests the role of blood brain barrier (BBB) injuries. Differentially expressed genes (DEGs) meta-analysis and protein–protein interaction (PPI) network analysis suggested potential key genes involved in AD development that might be therapeutic targets. In conclusion, our systematical network analysis on published AD expression profiling data suggests the immunopathogenesis of AD and identifies key brain regions and genes.

## Introduction

As the most common form of dementia, Alzheimer’s disease (AD) is a major public health concern. The occurrence and development of AD is a continuous clinical and pathophysiological process, and the National Institute of Aging and Alzheimer’s Association (NIA-AA) research framework categorizes AD into three continuous stages: preclinical, mild cognitive impairment (MCI), and dementia (Albert et al., 2011; McKhann et al., 2011; Sperling et al., 2011). A large number of studies have pointed out that in AD, changes in molecular biological processes and brain function networks often appear before clinical symptoms, brain metabolic homeostasis, such as nerve growth factor metabolic pathway is impaired before clinical AD; a substantial proportion of nondemented older adults have amyloid-beta accumulation and amyloid plaque; lower functional connectivity was observed before cognitive changes by using resting-state MRI (Price et al., 2009; Sperling et al., 2011; Buckley et al., 2017; Pentz et al., 2020), but the detailed underlying mechanism is still unclear.

Brain transcriptome analysis is considered a powerful method for studying AD mechanisms, and many studies conducted to date have focused on the expression profiling of postmortem brain tissue from AD patients and controls (Blalock et al., 2004; Mirnics et al., 2005; Haroutunian et al., 2009; Kim et al., 2012; Zhang et al., 2013). In addition to finding differentially expressed genes (DEGs) that are significantly changed in AD patients, expression profiling can also provide more evidence about the systematic molecular processes underlying the etio-pathogenesis of AD. Based upon the associations between coexpressed gene modules and AD traits, several previous studies identified AD-related gene modules, which suggests that the biological processes that these genes contribute to may be affected in AD (Wang et al., 2016; Tang and Liu, 2019; Hu et al., 2020; Kelly et al., 2020). By using spatial-temporal expression pattern analysis, transcriptome data can also provide evidence about specific brain regions and cell types that are possibly related to AD (Wang et al., 2016). It has been reported that the spatial-temporal pattern of gene expression in the brain shows strong correspondence with brain function (Richiardi et al., 2015; Anderson et al., 2018), so expression profiling of AD patients may provide more information about brain functional changes during AD development.

In the current study, we used published AD expression profiling data to construct 57 spatiotemporal specific (19 brain regions by three disease stages) coexpression networks in AD. By analysing the network preservation features of each brain region in different disease stages, the most dramatically changed coexpression modules were identified. Based on these modules, AD-related biological pathways, brain regions and circuits, cell types and key genes were analyzed.

## Data and Methods

### Gene Expression Profiling and Data Normalization

Dataset GSE84422 was downloaded from the National Biotechnology Information Center (NCBI) comprehensive gene expression database.^1^ This dataset contains the expression data of 1,054 brain tissue samples distributed in 19 brain regions of 125 subjects (Wang et al., 2016). Based on their Clinical Dementia Rate (CDR) score, subjects in GSE84422 were divided into three groups: control group, CDR 0–0.5; mild group, CDR 1–2, severe group, CDR 3–5 (as shown in Table 1). The raw microarray data were preprocessed by using RMA with quantile normalization (Irizarry et al., 2003). According to different platforms, hgu133a.db, hgu133b.db, and hgu133plus2.db are used for ID conversion. The average expression value of the probe set for each gene was used as its expression value.

**TABLE 1:** Brain regions and disease groups information of samples used in co-expression analysis.

Region	Full region name	Microarray Platform	Control	Mild	Severe
AC	Anterior cingulate	Affy 133 A and B	23	18	18
AMY	Amygdala	Affy 133Plus2	17	12	22
CN	Caudate nucleus	Affy 133 A and B	15	16	21
DPC	Dorsolateral prefrontal cortex	Affy 133 A and B	24	16	17
FP	Frontal pole	Affy 133 A and B	21	17	25
HIPP	Hippocampus	Affy 133 A and B	17	17	21
IFG	Inferior frontal gyrus	Affy 133 A and B	18	17	18
ITG	Inferior temporal gyrus	Affy 133 A and B	20	18	20
MTG	Middle temporal gyrus	Affy 133 A and B	22	14	22
NAC	Nucleus accumbens	Affy 133Plus2	17	12	22
OVC	Occipital visual cortex	Affy 133 A and B	21	18	14
PCC	Posterior cingulate cortex	Affy 133 A and B	18	15	24
PCG	Parahippocampal gyrus	Affy 133 A and B	15	14	20
STG	Superior temporal gyrus	Affy 133 A and B	17	19	20
PG	Precentral gyrus	Affy 133 A and B	21	20	19
PUT	Putamen	Affy 133 A and B	16	18	18
SFG	Superior frontal gyrus	Affy 133 A and B	23	18	19
SPL	Superior parietal lobule	Affy 133 A and B	20	14	16
TP	Temporal pole	Affy 133 A and B	19	16	23

### Coexpression Network in Different Brain Regions

Weighted gene coexpression network analysis (WGCNA) was performed on dataset GSE84422 to identify the gene modules with coordinated expression patterns for each brain region in different disease severities (Zhang and Horvath, 2005). After data normalization, the top 25% of the expressed genes in each brain region at each disease stage were taken as input genes, and coexpression networks were constructed using the R package WGCNA (Langfelder and Horvath, 2008). Briefly, Pearson’s correlation coefficients were calculated between all pairs of genes after microarray data normalization. Next, the correlation matrix was converted into an adjacency matrix using a power function f(x) = xβ, where x was the element of the correlation matrix and parameter β was determined such that the resulting adjacency matrix was approximately scale-free (Zhang and Horvath, 2005). The appropriate power value was estimated by a gradient test (power value ranging from 1 to 20) and determined when the scale independence value was equal to 0.85. The adjacency matrix was subsequently transformed into a topological overlap matrix (TOM), which captured both the direct and indirect interactions between each pair of genes (Ravasz et al., 2002). Average linkage hierarchical clustering was then employed to cluster the genes based on the TOM. Finally, a tree cutting algorithm was used to dynamically cut the hierarchical clustering dendrogram branches into highly connected modules, each of which was assigned a distinct colour code.

### Module Preservation Analysis

For each brain region, the preservation of coexpression modules across different disease stages was analyzed by using the R package NetRep (Ritchie et al., 2016). Coexpression networks of the control group were used as the discovery dataset, networks of the mild and severe groups were regarded as the tested datasets, and 10,000 permutations were performed. The NetRep statistics module preservation using seven statistical test methods, as recommended by the software, was applied. Modules whose *P*-value was less than 0.0001 in all seven methods were identified as strong preservation modules; those with *P*-values less 0.0001 in 1–6 methods were identified as weak preservation methods; and those with *P*-values not less than 0.0001 in any method were identified as non-preservation modules(NPMs).

### Functional Enrichment Analysis

To identify the biological processes in which NPM genes are involved, the Cytoscape plug-in ClueGO genes were used to provide a system-wide view (Bindea et al., 2009). The set including all NPM genes was used as the input gene set, and the ClueGo parameters were set as indicated: GO biological process, cellular component, and molecular function terms; display pathways with *P*-values ≤0.05; GO tree interval, three min level and eight max level; GO term minimum # genes, 5; threshold of 10% of genes per pathway; and a kappa score of 0.9. Pathway *P*-values were adjusted with Benjamini-Hochberg to 0.0100. The pathways were then represented, taking advantage of Cytoscape’s complex visualization environment as kappa score-based functional groups and named by the most significant term of each group. For each NPM, a specific pathway cluster enrichment analysis was performed by using the online analysis tool DAVID (Huang da et al., 2009). As recommended in DAVID, the cut-off for pathway cluster enrichment was set at a score > 1.3. The representative biological terms associated with significant clusters were manually selected.

### 3-D Brain Region Module

3-D modules for brain regions affected by NPMs were formulated by using Mango image processing software (Lancaster, Martinez).^2^ The labels of brain regions were obtained from the Talairach Atlas^3^ (Lancaster et al., 2000).

### Cell-Type Enrichment Analysis

Cell-type enrichment analyses of NPM genes were performed with the web-based tool Brain Expression Spatio-Temporal pattern (BEST) in http://best.psych.ac.cn (Guo et al., 2019). Cell type-specific expression profiles, which provide specific expression gene sets for astrocytes, endothelial cells, microglia, neurons, and oligodendrocytes, were obtained from http://www.brainrnaseq.org (Zhang et al., 2016). Fisher exact tests (FETs) were performed between each NPM and cell type, and the negative logarithm of the FET *P*-value was defined as the enrichment score.

### Meta-Analysis of DEGs

Gene expression studies of AD that utilized tissue samples from the middle temporal gyrus (MTG) and total temporal cortex (TC) were searched in GEO by keyword searches and manual selection. In total, six datasets were selected for meta-analysis: GSE132903, GSE5281, and GSE84422 for MTG; GSE131617, GSE36980, and GSE118553 for TC. Sample statuses in different studies are heterogeneous, so only data of defined controls and AD patients were used, and data from patients with probable AD and other diseases were excluded. The sample information is summarized in Table 3. Data quality control and meta-analysis were performed with the online tool ImaGEO^4^ (Toro-Dominguez et al., 2019). The maximum *P*-value method was selected, allowed missing values (%) was set at 10, and the adjusted *P*-value threshold was set at 0.05.

### Protein–Protein Interaction Analysis

DEGs and NPM genes in MTG were combined in the MTG gene set; DEGs in TC and NPM genes in ITG, MTG, and STG were combined in the TC gene set. The two gene sets were used as input data to perform protein–protein interaction (PPI) analysis in the online PPI network analysis platform STRING (Szklarczyk et al., 2019). The full STRING network was used to generate the MTG and TC networks by adding evidence edges between the input genes, and the minimum required interaction score of edges was 0.4. The generated networks were imported into Cytoscape, and the topological properties of the nodes were calculated using the plug-in “Network Analyzer” (Shannon et al., 2003; Assenov et al., 2008).

## Results

### 57 Coexpression Gene Networks of 19 Brain Regions in Three Different Disease Stages

**FIGURE 1:**
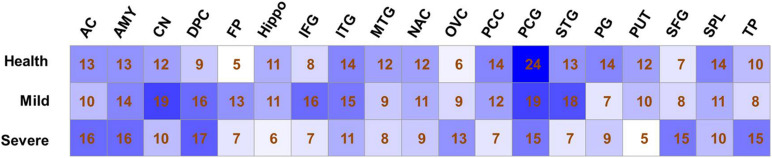
Modules number of co-expression networks in different sample sets. The number of co-expression modules that identified by Weighted gene coexpression network analysis analysis is shown for each sample set.

According to the topological structure of the coexpression network, the differences in networks with different organizations can be compared to analyze the spatial distribution of a disease. Therefore, we divided the gene set selected from 19 brain regions into 57 expression matrices according to the different brain regions and disease degrees, an unbiased gene coexpression network of expression matrices was constructed, and coexpression modules were identified. The selection of the soft threshold and clustering results of each coexpression network can be seen in Supplementary Figure 1. The numbers of coexpression modules in each network are shown in Figure 1.

### Evidence of Preservation of the Coexpression Network in 19 Brain Regions

**FIGURE 2:**
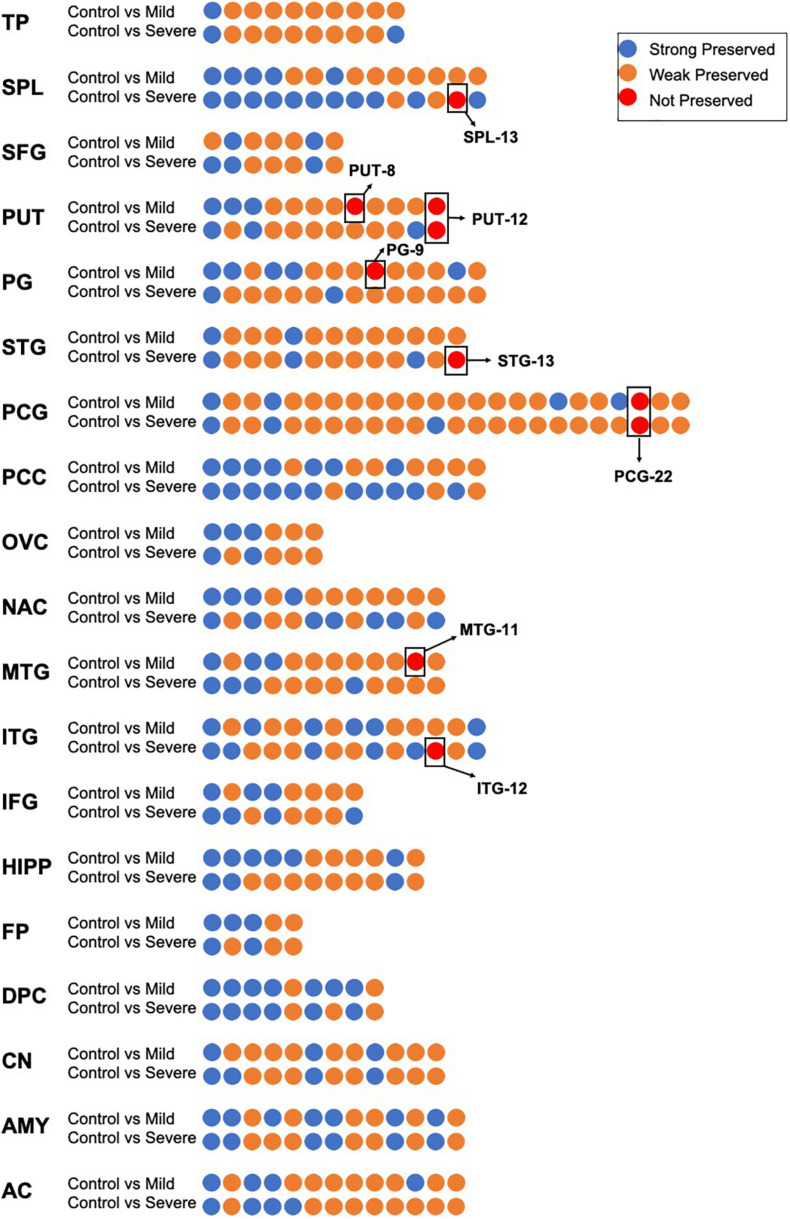
The preservation evidence of control co-expression modules in different disease stages and different brain regions. For each brain region, co-expression networks in control group was compared with networks in mild and severe groups, co-expression modules are show as dots, which are sorted according to module size and colored according to their preservation evidence. Blue: strong preservation evidence; yellow: weak preservation evidence; red: none preservation evidence.

As shown in Figure 2, in the control groups, there were 233 coexpression modules in 19 brain regions; among them, 164 were less preserved in the process of AD, accounting for 73.54% of the total. Furthermore, eight modules in seven brain regions were identified as not preserved. Three modules were non-preserved in mild AD: the 12th in ITG (ITG-12), the 13th in SPL (SPL-13), and the 13th in STG (STG-13). Three modules were not preserved in severe AD: the 11th module in MTG (MTG-11), the 8th module in PUT (PUT-8) and the 9th module in PG (PG-9). Two modules are non-preserved in both mild and severe AD cases: the 12th module of the PUT brain regions (PUT-12) and the 22nd module of the PCG brain regions (PCG-22). Detailed gene lists of each NPM are shown in Supplementary Table 1.

### Functional Enrichment of the NPM Genes

**FIGURE 3:**
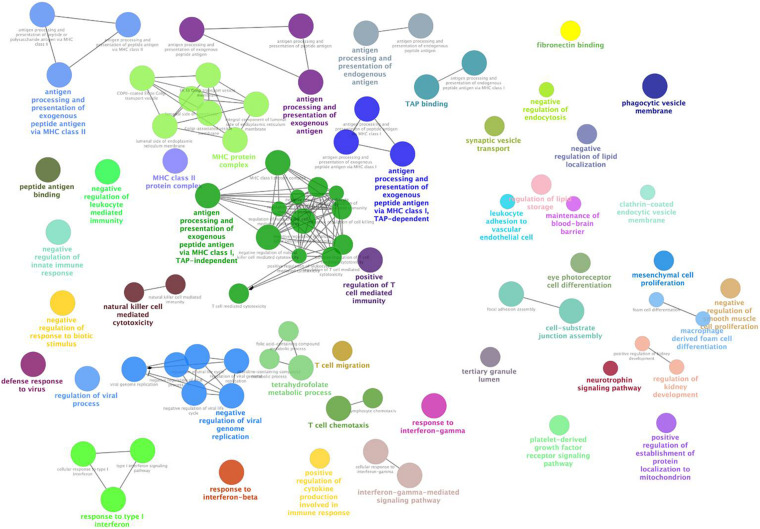
Functional GO groups enriched by genes in non-preserved co-expression modules. This figure illustrates the functionally grouped network that constructed by GO terms (nodes) that associated to non-preserved co-expression module genes. The size of the nodes reflects the statistical significance of the terms. The degree of connectivity between terms (edges) is calculated using kappa statistic and functional groups are defined using the kappa score. The name of the group is given by the most significant term in the group and nodes in the same group are represented with same color.

We observed the functional distribution of the NPM genes by GO term network analysis using ClueGo. Finally, these genes were found to be enriched in 44 GO term groups (constructed by 85 GO terms). As shown in Figure 3, most of the enriched GO groups were related to the immune response, and functions related to cell differentiation, vesicle transport, and lipid metabolism were also involved. Functional enrichment analyses were also performed for genes in each NPM, as shown in Table 2. Genes in four NPMs were significantly enriched in the functional pathway clusters, and the enriched clusters were mainly related to the immune response.

**TABLE 2:** Pathway clusters significantly enriched by genes of non-preservation modules (enrichment score > 1.3).

Non-preservation modules	Annotation cluster	Representative annotation terms	Enrichment score
ITG-12	1	Immune response	1.69
SPL-13	None		
STG-13	None		
MTG-11	None		
PUT-8	1	Defense response to virus	10.78
	2	Virus infection	1.85
	3	Proteolysis involved in cellular protein catabolic process	1.51
	4	Hydrolase	1.4
PG-9	1	Defense response to virus	5.76
	2	Interferon-gamma-mediated signaling pathway	2.04
	3	Negative regulation of transcription from RNA polymerase II promoter	1.90
	4	CUB domain	1.79
	5	Golgi apparatus	1.66
	6	EGF-like domain	1.56
	7	Complement pathway	1.43
PUT-12	None		
PCG-22	1	Cholesterol metabolism	1.48

**TABLE 3:** Datasets used in the meta-analysis.

Brain region	dataset	cases	controls	DEGs	DEGs after meta-analysis
MTG	GSE132903	97	98	6,908	192
	GSE5281	16	12	2,253	
	GSE84422	20	14	0	
Temporal cortex	GSE131617	58	13	0	62
	GSE36980	10	19	258	
	GSE118553	45	26	2,475	

### Brain Region Distribution of Non-preservation

**FIGURE 4:**
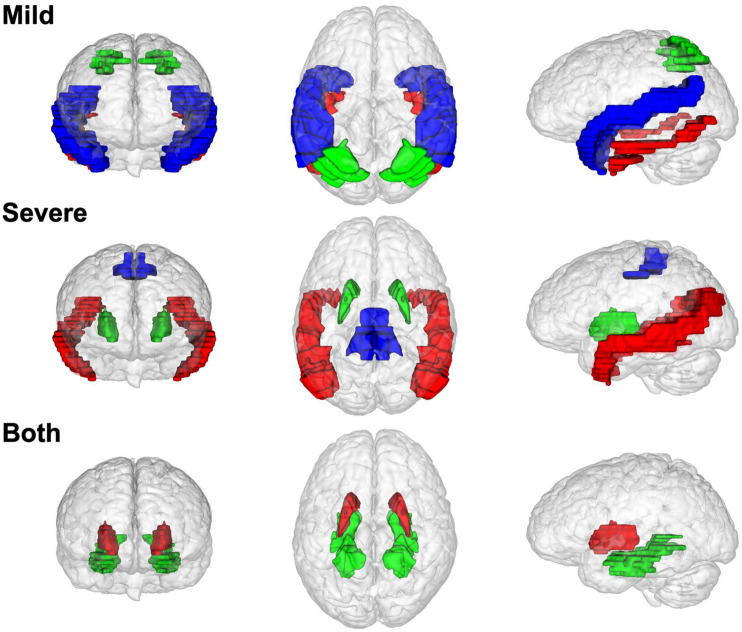
The brain region distribution of non-preserved modules in different Alzheimer’s disease stages. Modules that non-preserved in mild stages are located in superior temporal gyrus (blue), inferior temporal gyrus (red), and superior parietal lobule (red). Modules that non-preserved in severe stage are located in middle temporal gyrus (red), putamen (green), precentral gyrus (blue). Modules that non-preserved in both two disease stages are located in putamen (red) and parahippocampal gyrus (green).

Figure 4 shows the brain region distribution of the NPMs in different disease stages. NPMs in mild stages are located in two gyri of the temporal cortex (superior temporal gyrus, inferior temporal gyrus) and a lobule of the parietal cortex (superior parietal lobule). NPMs in the severe stage are located in the temporal cortex (middle temporal gyrus), primary motor cortex (precentral gyrus), and basal nuclei (putamen). NPMs in both disease stages are located in the basal nuclei (putamen) and limbic system (parahippocampal gyrus).

### Cell-Type Enrichment of NPM Genes

**FIGURE 5:**
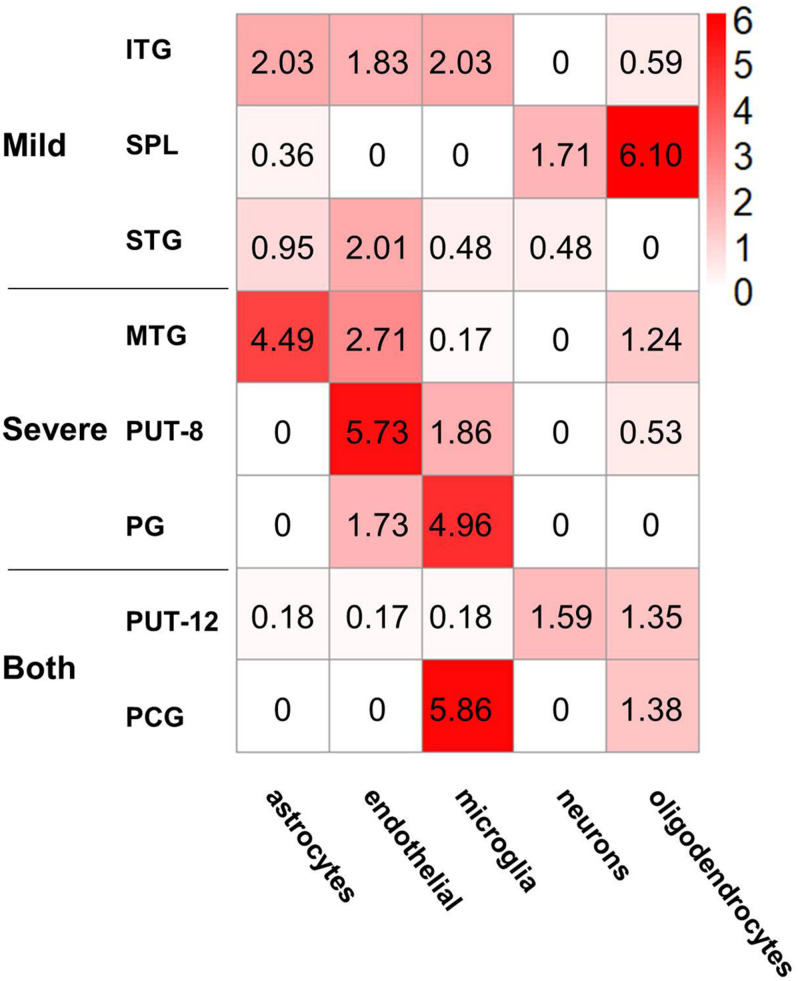
Cell type enrichment of non-preserved modules in different brain regions. The heatmap shows the enrichment results of five kinds of cell type-specific genes in the eight non-preserved modules, the color in heatmap illustrated the negative logarithm of P-value of the enrichment, from white (-log(*P*-value) = 0) to red (-log(*P*-value) > 6). The significant cutoff is defined as 1.3, which is equivalent to *P*-value 0.05.

Gene sets associated with five kinds of brain cells were used in enrichment analysis of genes in each NPM. Figure 5 shows the enrichment scores of the NPMs in different cell types. According to the cut-off of 1.3 (equivalent to a *P*-value of 0.05 in FET), 2 modules were significantly enriched in astrocytes, 5 in endothelial cells, 4 in microglia, 2 in neurons, and 3 in oligodendrocytes. Strong significance appears in astrocytes, endothelial cells, microglia, and oligodendrocytes.

### PPI Network of DEGs and NPM Genes in MTG and TC

**FIGURE 6:**
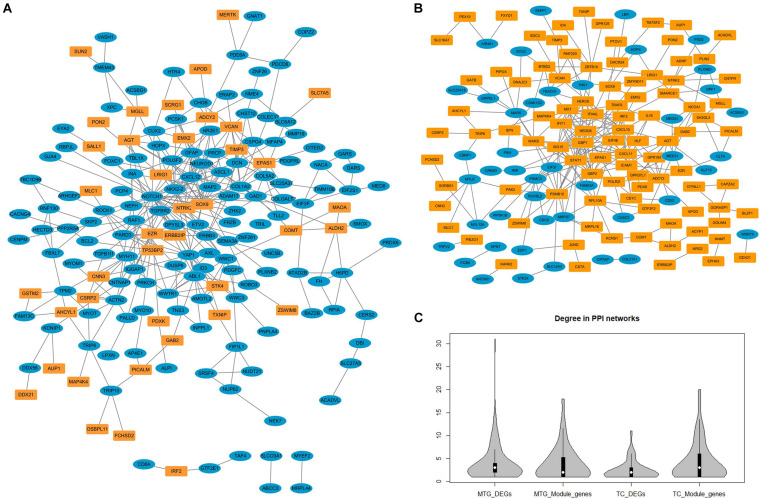
The protein–protein interaction networks constructed by differentially expressed genes and genes in non-preserved modules. **(A)** Protein-protein interaction network constructed by middle temporal gyrus differentially expressed genes (blue nodes) and non-preserved module genes (yellow nodes). **(B)** Protein–protein interaction network constructed by temporal cortex differentially expressed genes (blue nodes) and non-preserved module genes (yellow nodes). **(C)** The degree distribution of different types of genes in protein-protein interaction network.

Since all three temporal gyri are involved in disease development, meta-analyses were performed to identify DEGs in the temporal cortex or specific temporal gyrus. Finally, six data sets were selected, and the results of the meta-analyses are summarized in Table 3. Detailed information on the meta-analysis is shown in Supplementary Tables 2, 3 and Supplementary Figures 2–5. No overlap was observed between the DEGs and NPM genes, but there were universal PPIs among them (as shown in Figures 6A,B). As shown in Table 4, 177 of 241 MTG genes (including DEGs and NPM genes) can be found in STRING, 342 interactions between them can be found, significantly (*P* = 2.83E-14) higher than the predicted interaction number 222, predicted by the average interaction number in the STRING network. A total of 141 of 207 TC genes were found in STRING, and 262 interactions were found among them, which was significantly (*P* = 0.00145) higher than the predicted number of 216. The average degree, degree distribution, and high degree genes (degree ≥ 10) are shown in Table 4 and Figure 6C.

**TABLE 4:** Summary of nodes in the PPI networks of MTG and TC.

	MTG				TC			
	DEGs		NPM genes		DEGs		NPM genes	
Total number	192		49		62		145	
Numbers in PPI networks	137		40		44		97	
Average degree	3.88		3.88		2.41		4.3	

**High degree genes (degree ≥ 10)**	**Gene symbol**	**Degree**	**Gene symbol**	**Degree**	**Gene symbol**	**Degree**	**Gene symbol**	**Degree**

	NOTCH1	31	SOX9	18	PSMC3	11	ISG15	20
	GFAP	15	EZR	13			CXCL10	18
	YAP1	14	NTRK2	11			STAT1	18
	CXCL12	13	VCAN	10			GBP1	14
	NEUROD1	12					CXCL11	13
	DCN	11					PSMB10	12
	ASCL1	11					GBP2	12
	ABL1	11					ICAM1	11
	COL1A2	10					MX1	11
	MYH11	10					NEDD8	11
							AGT	10
							HERC6	10
							IFIT1	10

## Discussion

In the current study, we explored AD-related biological processes by analysing the coexpression gene modules of different brain regions in different disease stages. The coexpression network features and key genes of AD peripheral blood or brain have been reported in several previous studies (Seyfried et al., 2017; Liang et al., 2018; Sweeney et al., 2018; Zhang et al., 2018; Hu et al., 2020; Kelly et al., 2020; Soleimani Zakeri et al., 2020). Among these studies, Wang et al. performed a pan-cortical brain region genomic analysis, obtained and ranked 44,692 gene probesets, 1,558 coexpressed gene modules and 19 brain regions based upon their association with AD; through these analyses temporal lobe gyri were identified as sites associated with the greatest and earliest gene expression abnormalities, abnormal expression was specific to cell type of oligodendrocytes, astrocytes, and neurons, and neurobiological pathways (included actin cytoskeleton, axon guidance, and nervous system development) were enriched by abnormally expressed genes and modules (Wang et al., 2016); however, the changes in coexpression modules in sub-brain regions during AD development have not been fully studied. We constructed 57 coexpression networks by using this expression dataset from 1,053 postmortem brain samples across 19 cortical regions, evaluated network conservation during disease pathology (from healthy to mild and severe AD stages) in each brain region, and deduced disease-related biological processes based on the network features.

As we expected, in the development of AD, there is a wide range of coexpression pattern changes in the whole brain. This suggested that dysfunctions of expression appear in multiple brain regions, not only in brain regions that are traditionally associated with memory. We focused on the eight most dramatically changed coexpression modules. Functional pathway analysis suggested that genes in these modules are mainly involved in the immune response instead of transmitters or other pathways that directly affect neuronal function. These results supported the neuroimmunopathogenesis of AD. In recent years, AD has no longer been considered a neural-centric disease, and the critical role played by neuroinflammation in the pathogenesis of AD has been implicated in many genetic, functional, and neuroimaging studies (Cao and Zheng, 2018; Jansen et al., 2019; Kunkle et al., 2019; Passamonti et al., 2019; Bis et al., 2020; Burgaletto et al., 2020).

The most dramatic coexpression pattern changes occurred in seven brain regions. These results are consistent with previous brain structure or functional studies. Coexpression patterns in five regions began to change dramatically in the mild stage, including two subregions of the temporal cortex, one lobule of the parietal cortex, part of the dorsal striatum, and the parahippocampal gyrus in the limbic system. The associations of structural or functional changes in such brain regions and early AD pathology have been widely reported. It has been reported that tau pathology spreads hierarchically from the inferior temporal lobe throughout the cortex (Franzmeier et al., 2019), and neural activity increases in the superior parietal lobule of patients with MCI (Jacobs et al., 2012). Associations between dysfunction of the limbic system or basal ganglia and early AD have also been reported (Hopper and Vogel, 1976; Nestor et al., 2003; de Jong et al., 2008; Botzung et al., 2019). In severe AD, dysfunction spreads to the middle temporal gyrus and precentral gyrus. The relationship between the middle temporal gyrus and AD has been emphasized in many studies (Galton et al., 2001b; Dong et al., 2021). Although it has not been fully studied, the relationship between the precentral gyrus and AD has been reported in several studies (Peters et al., 2009).

Considering the correlation between brain expression patterns and brain functional connections (Richiardi et al., 2015; Anderson et al., 2018), the expression changes suggested potential connectivity changes in AD, and the evidence in the current study corresponds to previous reports. Regional tau PET levels within major functional networks showed a medial temporal limbic network-specific distribution (Franzmeier et al., 2019), and limbic network and striatal connectivity alternated in patients with AD and MCI (Badhwar et al., 2017), and an increased effectiveness of temporoparietal connectivity has been reported in AD patients (Jacobs et al., 2012).

In addition to brain region distribution features, violent coexpression pattern changes present a cell type-specific distribution. In general, all NPM genes were most significantly enriched in non-neuron cells (astrocytes, endothelial cells, microglia, and oligodendrocytes), and only NPM genes in the SPL and putamen showed an enrichment trend in neurons. Considering the roles of glia in AD and the immune response (Gonzalez-Reyes et al., 2017; Fakhoury, 2018; Leng and Edison, 2021), the enrichment of astrocytes and microglia may highlight the immunopathology in AD. Oligodendrocytes are located in the white matter, recent neuroimaging studies have implicated micro- and macrostructural abnormalities in white matter in the risk and progression of AD (Nasrabady et al., 2018), it’s reported that age and severity of dementia were significantly associated with white matter changes in AD patients (Kao et al., 2019), and white matter may also play an important role in the pathogenesis and diagnosis of AD, besides of intact with gray matter, demyelination of the white matter is reported to occur prior to the presence of amyloid-β plaques and neurofibrillary tangles in the presymptomatic stages of AD (Sachdev et al., 2013). Oligodendrocytes may affect AD pathogenesis in both neuropathological and immunopathological manners, oligodendrocytes are regulated by Aβ oligomers in differentiation and maturation (Quintela-Lopez et al., 2019), oligodendrocyte precursor cells present antigens and may be involved in perpetuating the autoimmune response (Kirby et al., 2019). Endothelial cells participate in the formation of the blood brain barrier (BBB), and Aβ influences endothelial mitochondrial dysfunction pathways and contributes to the progression of neurovascular dysfunction in AD. The enrichment results in the current study also support BBB-related pathology in AD (Parodi-Rullan et al., 2019). Additionally, the functional enrichment analysis also suggested that the dysfunction of NPM genes influences the maintenance of the BBB.

The temporal lobe, especially the MTG, is an important brain area involved in cognition and memory (Kornblith et al., 2017; Naya et al., 2017; Vaz et al., 2019) and has been a focus in AD pathology (Galton et al., 2001a; Visser et al., 2002; Dickerson and Sperling, 2008; de Flores et al., 2020). Several genes have been reported to be differentially expressed in the temporal lobe between patients with AD and healthy controls, but the results of different studies are heterogeneous (Patel et al., 2019). In the current study, we searched transcriptomics data of MTG or TC and performed a meta-analysis. As shown in Table 3, articles in both groups presented high heterogeneity. When the cut-off of DEGs was set as a *P*-value less than 0.05 and a fold change of more than two or less than 0.5, both meta-analyses failed to identify DEGs. This may be due to the high degree of heterogeneity among the studies. When the cut-off was adjusted to a *P*-value less than 0.05, some stable but not violent DEGs were identified. None of these DEGs were in NPMs, but they were extensively connected to NPM genes in PPI networks. This suggests that AD pathology is not caused by drastic changes in a few genes but is related to changes in the entire expression pattern. A large number of genes were involved in the pattern change, but for a single gene, the change was not dramatic.

Network topology analysis showed that the degree of NPM genes was higher than that of DEGs, but this trend was not significant. According to the hypothesis that disease genes tend to have higher degrees in the network (Jonsson and Bates, 2006; Sun et al., 2010), high degree genes were identified and may play more important roles in the functional network and AD pathology. Several high degree genes have been reported to participate AD pathology related pathways or related to AD, *ABL1,SOX9, STAT1, PSMB10, NEDD8, HERC6*, and *IFIT1* have been reported as participants of amyloid- or Tau-signaling (Jing et al., 2009; Chen et al., 2012; Orre et al., 2013; Woodling et al., 2014; Li et al., 2019; Vong et al., 2021); *DCN, CXCL10, CXCL11*, and *ICAM1* play roles in amyloid plaque formation (Frohman et al., 1991; Snow et al., 1992; Krauthausen et al., 2015); *NOTCH1, GFAP, YAP1, CXCL10,CXCL12, ASCL1, STAT1, GBP1, GBP2*, and *AGT* are altered expression in plasma, CSF, or brain of AD patients (Kitamura et al., 1997; Galimberti et al., 2006; Laske et al., 2008; Mateos et al., 2011; Xu et al., 2018; Cho et al., 2019; Meyer et al., 2019; Oeckl et al., 2019; Kuan et al., 2021); AD association of variants in or surrounding *MYH11, NTRK2, PSMC3* and *ISG15* have been detected (Chen et al., 2008; Roy et al., 2020; Blue et al., 2021; Novikova et al., 2021). Relationships between AD and high degree genes *COL1A2, EZR*, and *VCAN* haven’t been reported, their roles in AD pathology need further study.

In conclusion, in the current study, we constructed 57 spatiotemporal specific coexpression networks in AD. By using network preservation analysis, we observed universal expression changes in all 19 brain regions. The eight most dramatically changed coexpression modules were identified in seven brain regions. Genes in these modules are mostly involved in immune response-related pathways, this supports the immune pathology of AD. The distribution of NPMs provides evidence of the brain functional mechanism of AD. The cell type distribution of NPMs also suggests the role played by the immune response and BBB injuries. In addition to revealing information about the potential etiopathogenesis of AD, our analysis suggested potential key genes involved in AD development that might be therapeutic targets.

Comprehensive analysis in this study provides new evidence for the immunopathological mechanism of AD, reveals the potential key brain regions, cells and molecular pathways in the development of AD. It provides new clues for the mechanism and intervention study of AD. In spite of above results, this study also has some limitations. Analysis in this study is based on public data, and the new results have not been verified in new samples or animal models, subsequent studies are needed to validate their stability. Our analyses only use expression data, genetic factors (such as APOE genotype) and demographic factors (such as gender and age) have not been considered, so the results should be further validated in more diverse populations. Although several high degree genes we identified have been reported altered expression in AD patients, more systematic validation and consequence functional researches should be performed to confirm their value.

## Data Availability Statement

The original contributions presented in the study are included in the article/Supplementary Material, further inquiries can be directed to the corresponding authors.

## Author Contributions

JW and LG designed the study and drafted the manuscript. LG and YL performed the analysis. All authors have read and approved the final manuscript.

## Conflict of Interest

The authors declare that the research was conducted in the absence of any commercial or financial relationships that could be construed as a potential conflict of interest.

## Publisher’s Note

All claims expressed in this article are solely those of the authors and do not necessarily represent those of their affiliated organizations, or those of the publisher, the editors and the reviewers. Any product that may be evaluated in this article, or claim that may be made by its manufacturer, is not guaranteed or endorsed by the publisher.

## Footnotes

1
https://www.ncbi.nlm.nih.gov/geo/

2
http://ric.uthscsa.edu/mango

3
http://www.talairach.org/

4
http://bioinfo.genyo.es/imageo/
