# Supplementary material for: Preservation Analysis on Spatiotemporal Specific Co-expression Networks Suggests the Immunopathogenesis of Alzheimer’s Disease
Source: Front Aging Neurosci. 2021 Sep 3;13:727928. doi: 10.3389/fnagi.2021.727928 (PMC8446362; doi:10.3389/fnagi.2021.727928)

Figure S1. Scale free network topology and cluster dendrogram of 57 spatiotemporal specific AD co-expression networks.

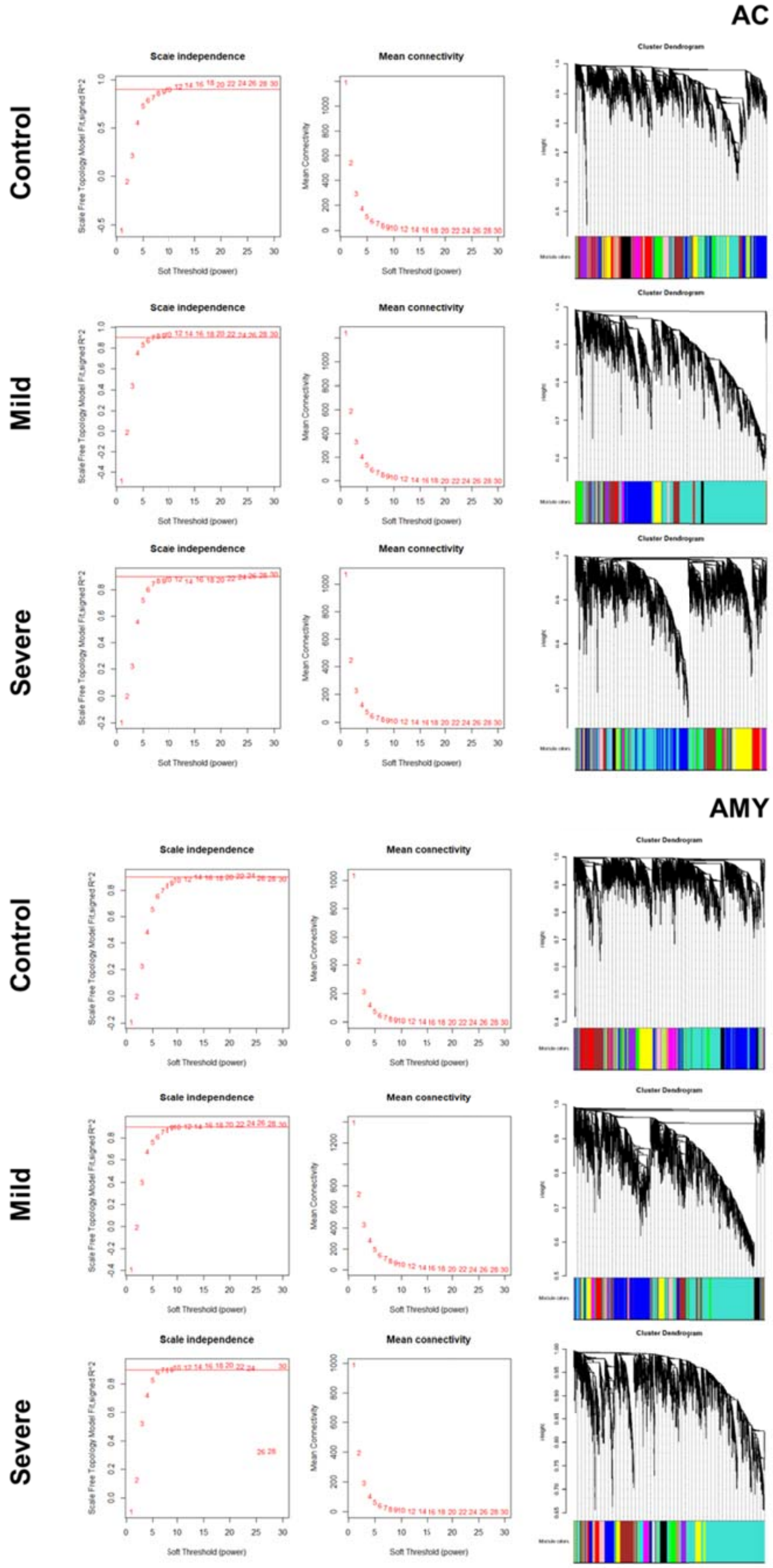

(Continued)

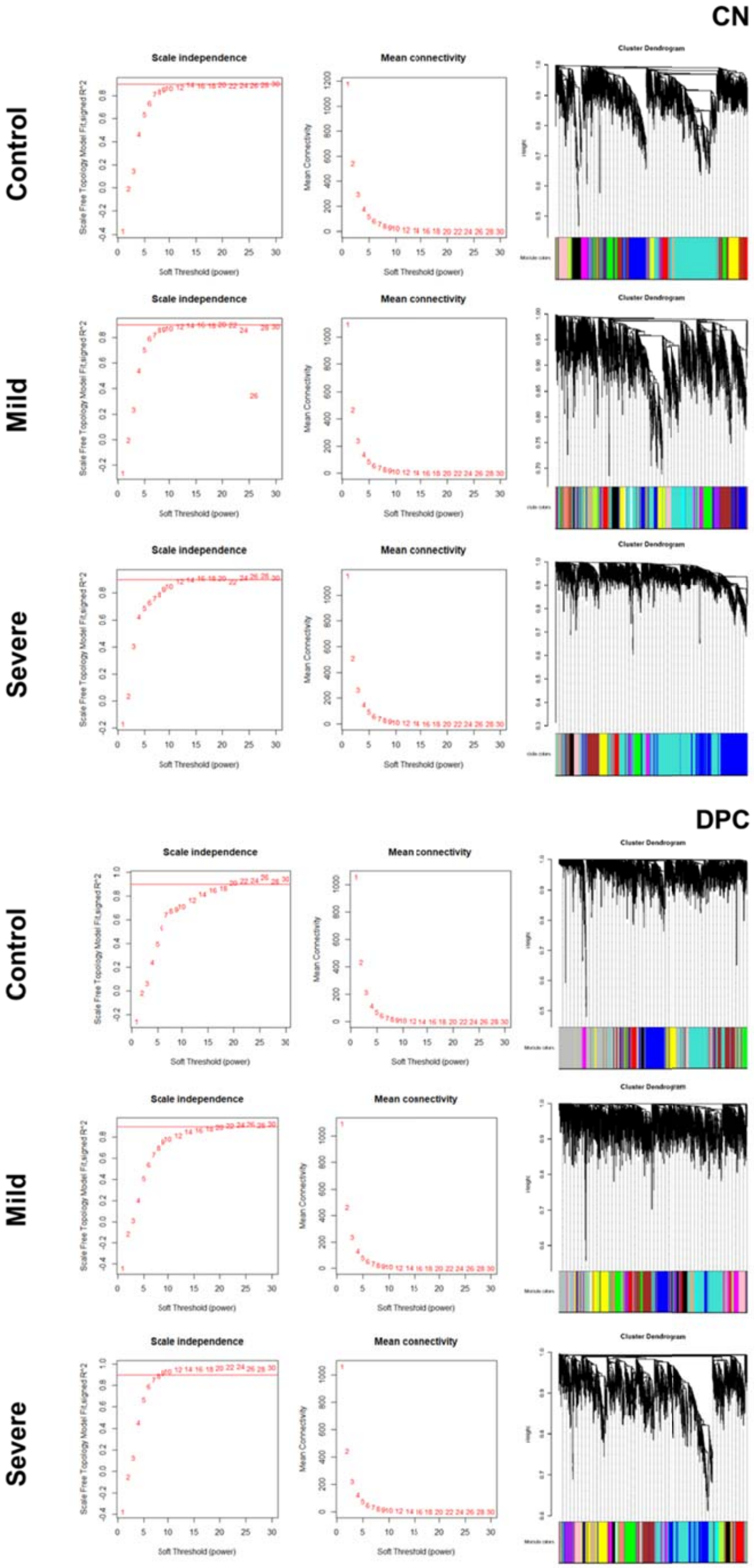
CN
Control
Mild
Severe
DPC
Control
Mild
Severe
Scale independence
Mean connectivity
Cluster Dendrogram
Scale Free Topology Model Fit,signed R^2
Mean Connectivity
Soft Threshold (power)
Height
Module colors

(Continued)

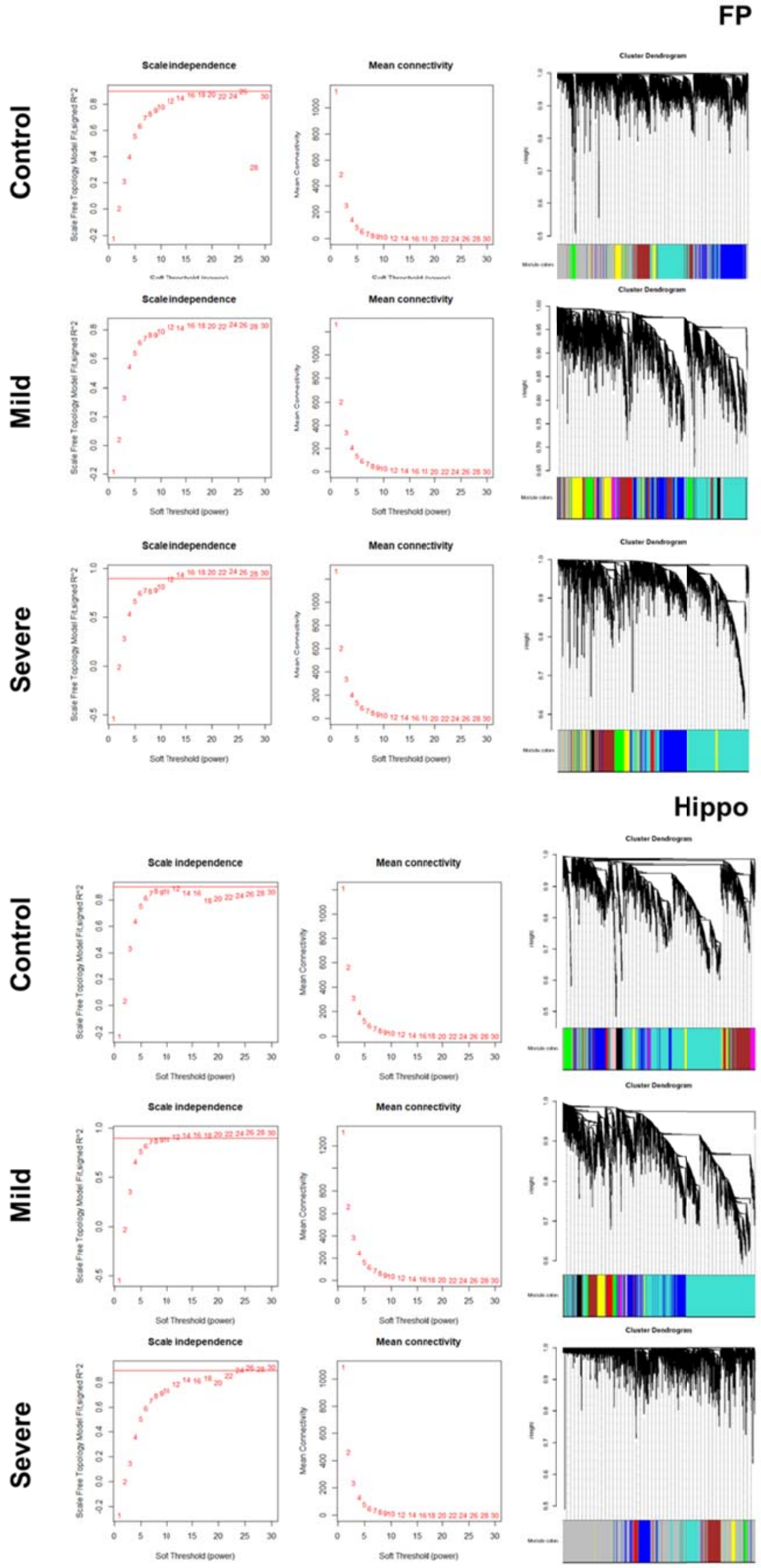

FP
Control
Mild
Severe
Hippo
Control
Mild
Severe
Scale independence
Mean connectivity
Cluster Dendrogram
Soft Threshold (power)
Scale Free Topology Model Fit,signed R^2
Mean Connectivity

(Continued)

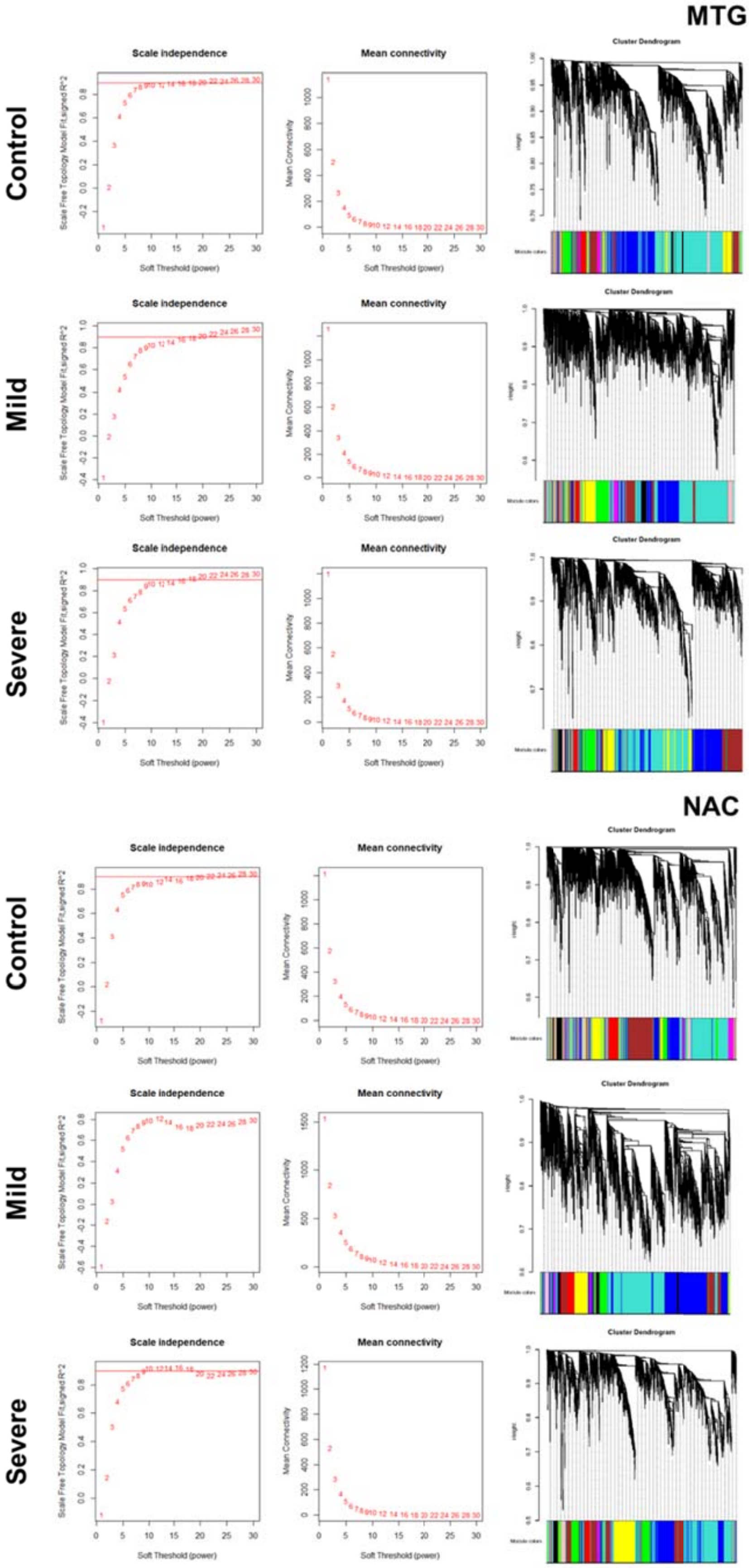

MTG
Control
Mild
Severe
NAC
Control
Mild
Severe
Scale independence
Mean connectivity
Cluster Dendrogram
Soft Threshold (power)
Scale Free Topology Model Fit,signed R^2
Mean Connectivity

(Continued)

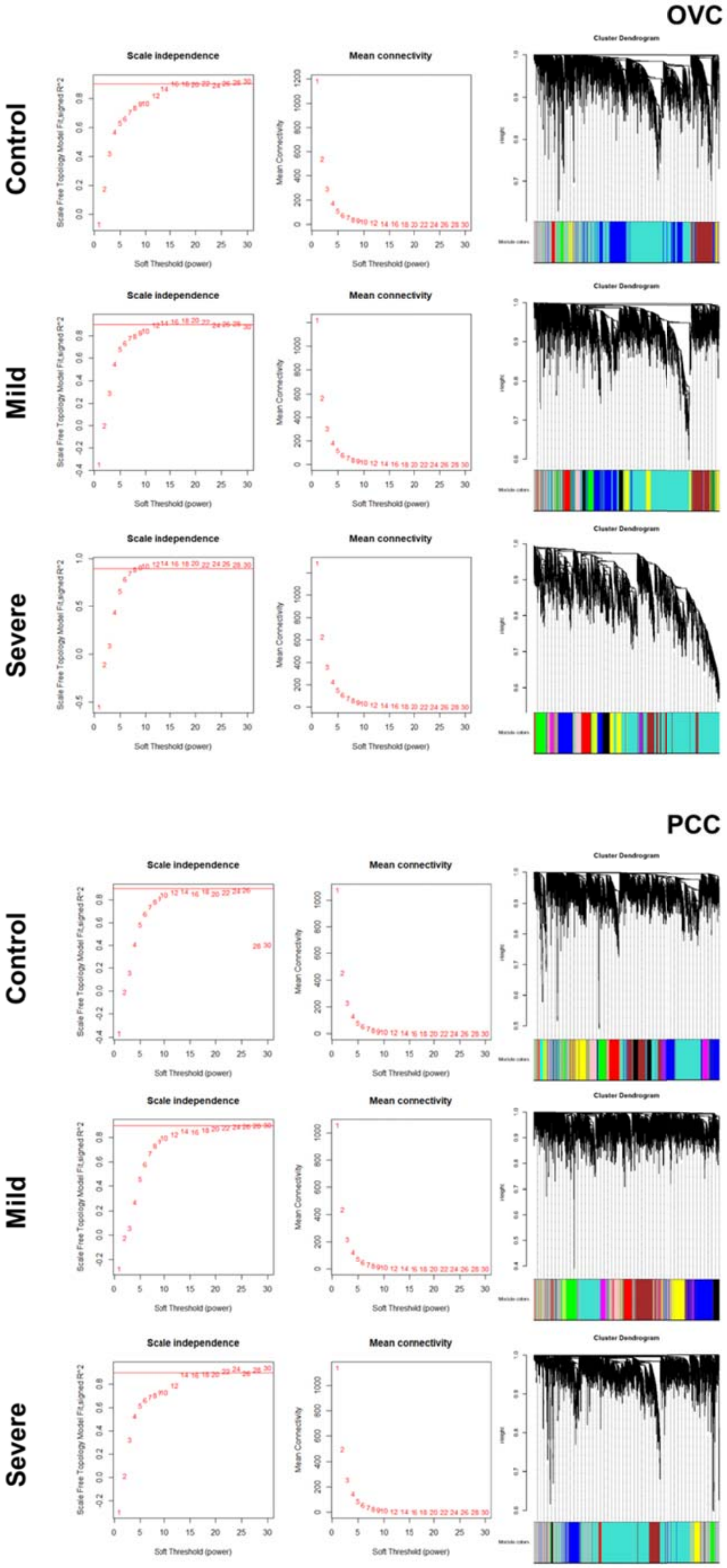
OVC
Control
Mild
Severe
Scale independence
Mean connectivity
Cluster Dendrogram
Soft Threshold (power)
Scale Free Topology Model Fit,signed R^2
Mean Connectivity
PCC
Control
Mild
Severe
Scale independence
Mean connectivity
Cluster Dendrogram
Soft Threshold (power)
Scale Free Topology Model Fit,signed R^2
Mean Connectivity

(Continued)

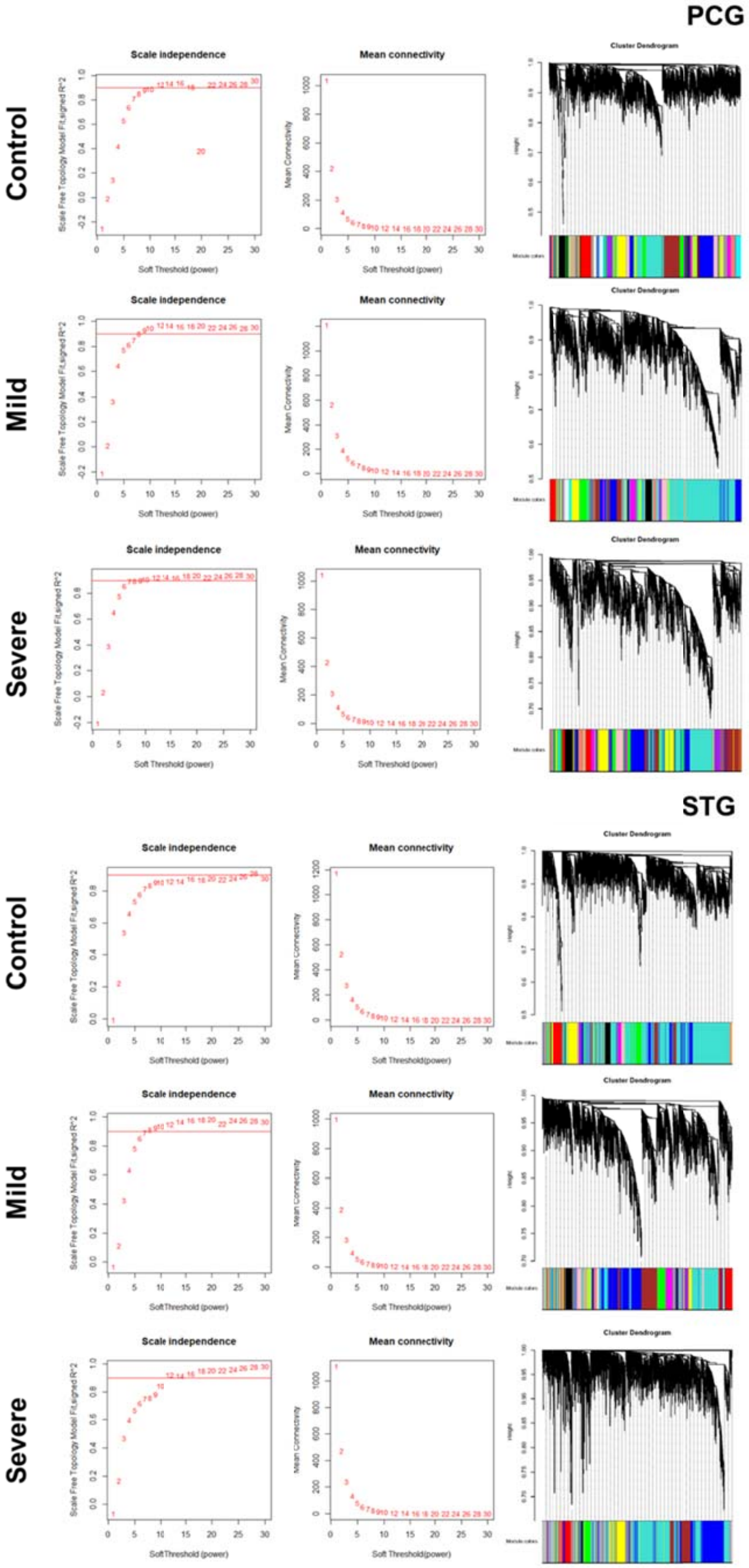
PCG
Control
Mild
Severe
Scale independence
Mean connectivity
Cluster Dendrogram
Soft Threshold (power)
STG

(Continued)

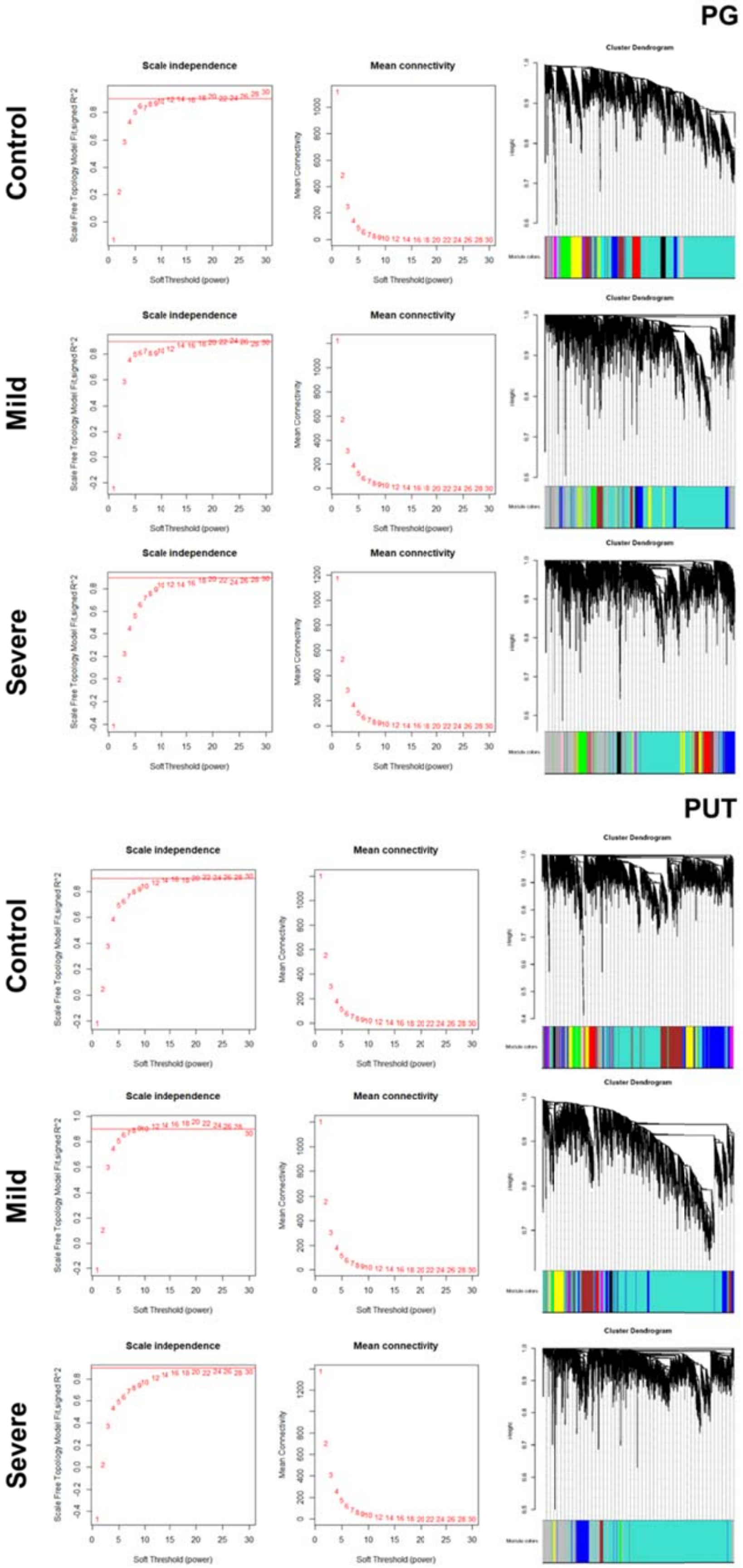
PG
Control
Mild
Severe
PUT
Control
Mild
Severe
Scale independence
Mean connectivity
Cluster Dendrogram
Soft Threshold (power)
Scale Free Topology Model Fit,signed R^2
Mean Connectivity

(Continued)

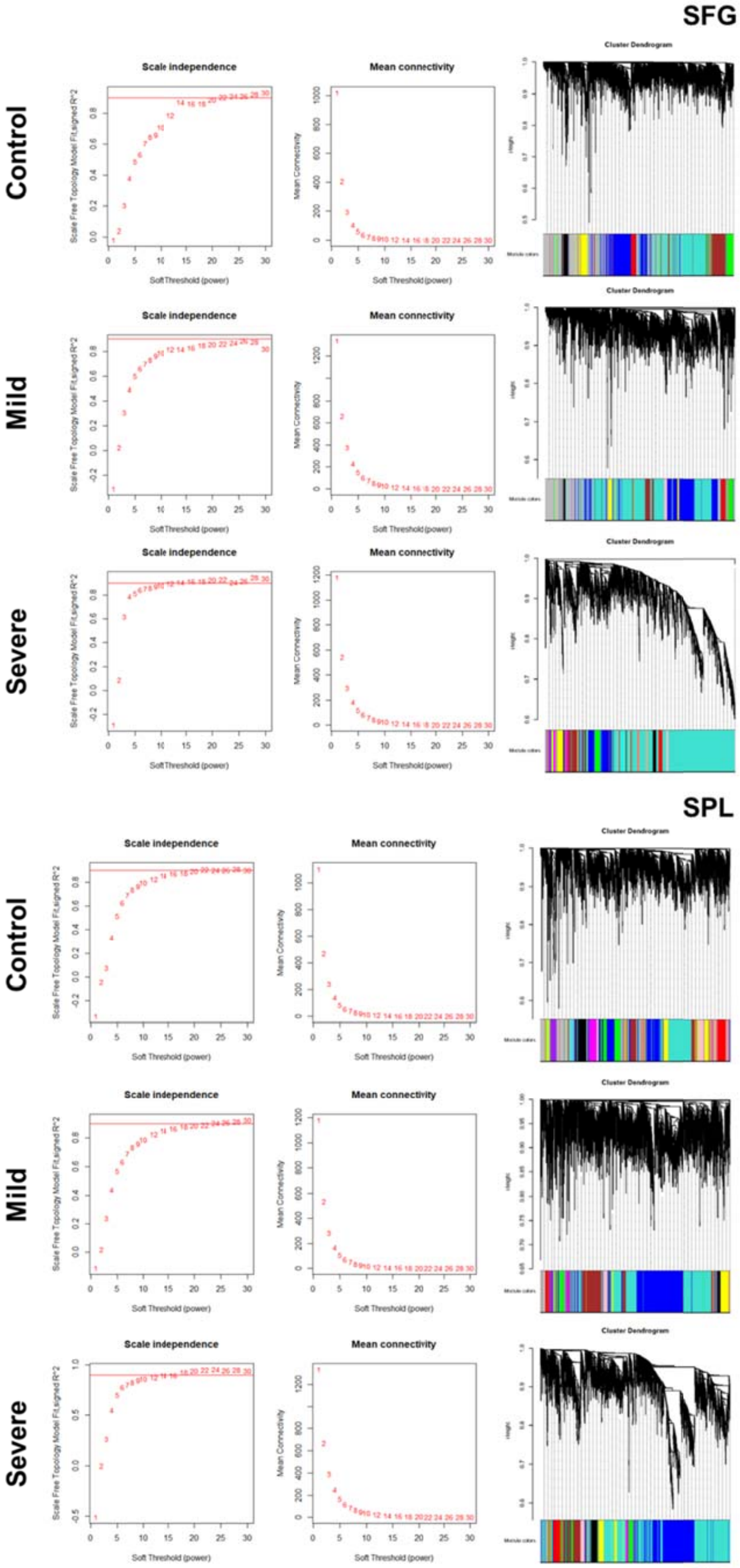
SFG
Control
Mild
Severe
Scale independence
Mean connectivity
Cluster Dendrogram
Soft Threshold (power)
Scale Free Topology Model Fit,signed R^2
Mean Connectivity
SPL
Control
Mild
Severe

(Continued)

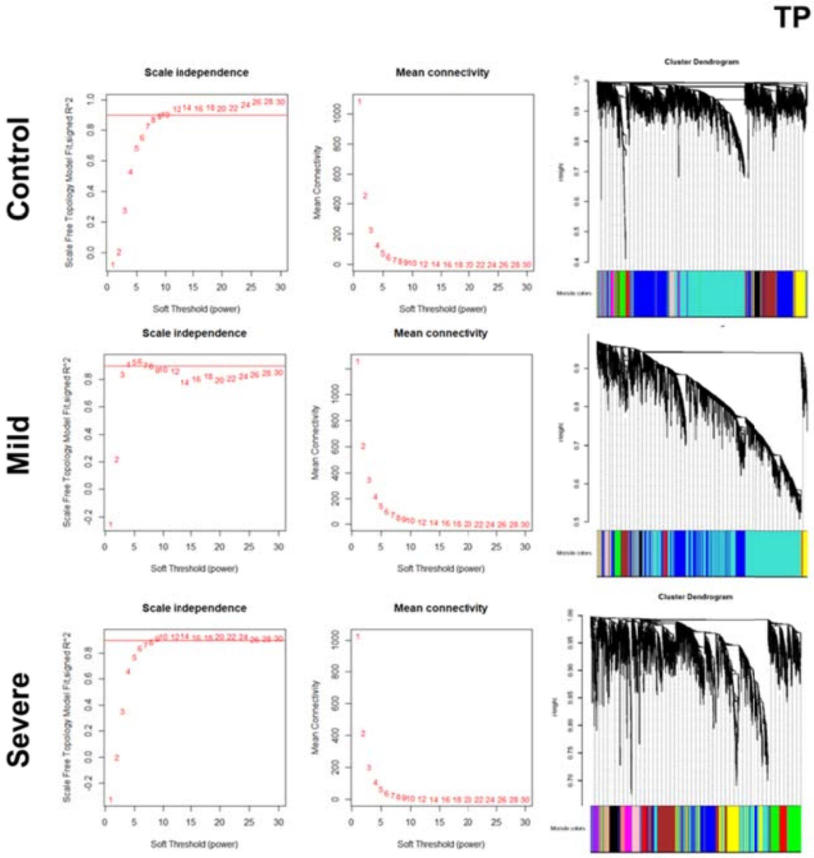
TP
Control
Mild
Severe
Scale independence
Mean connectivity
Cluster Dendrogram
Soft Threshold (power)
Scale Free Topology Model Fit,signed R^2
Mean Connectivity

Figure S2. Cassette figures of the MTG expression data after standardization

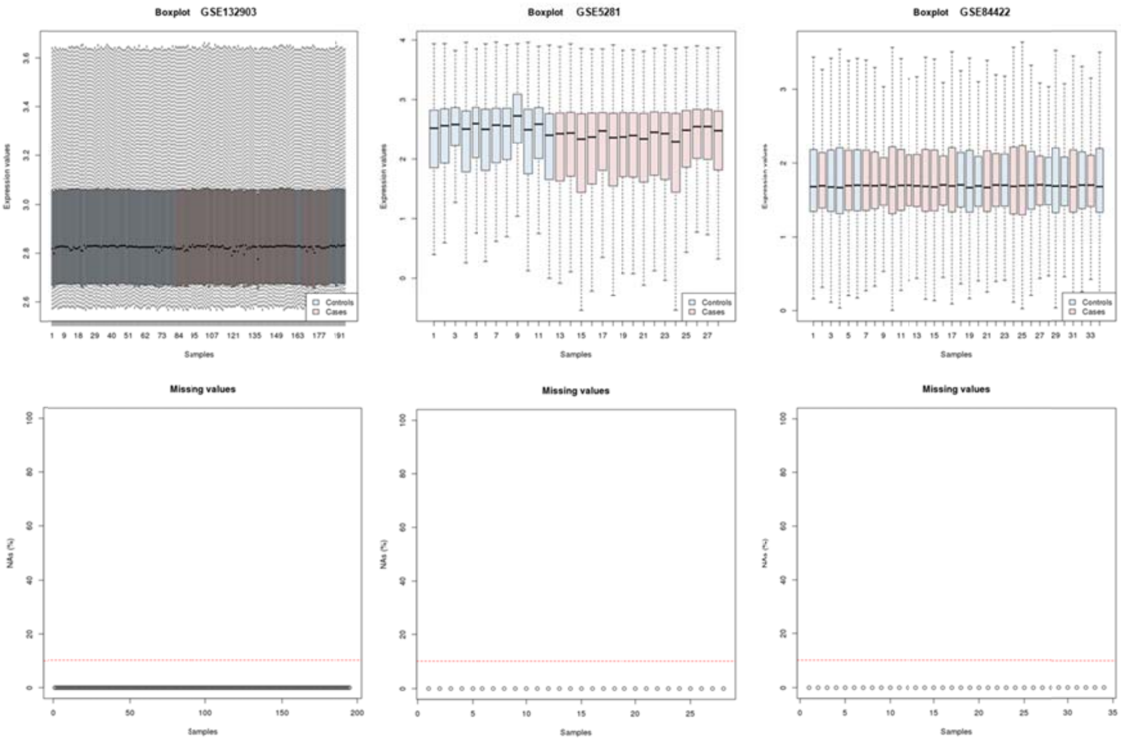

Figure S3. Heatmap of DEGs in MTG datasets.

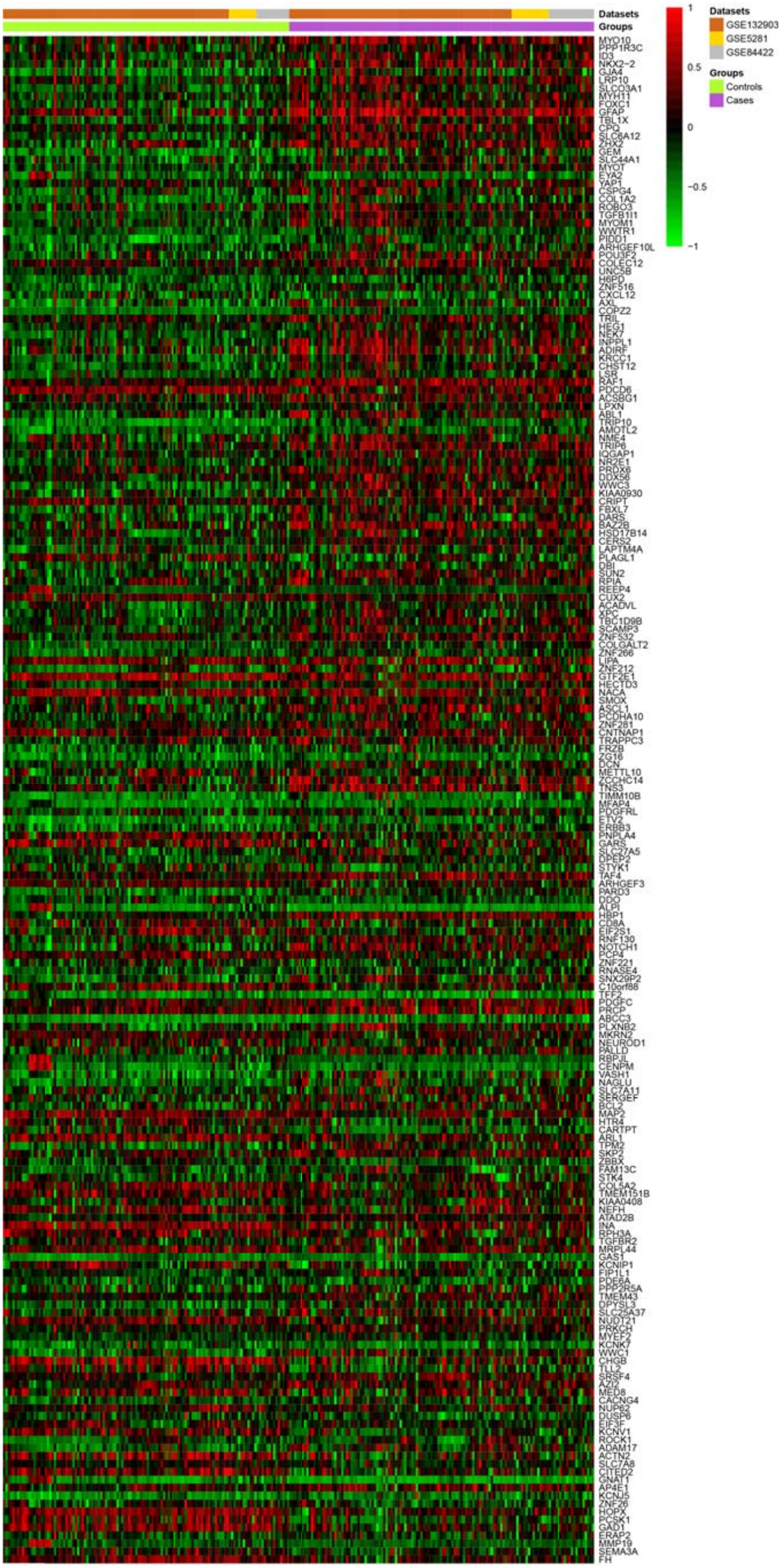

Figure S4. Cassette figures of the TC expression data after standardization

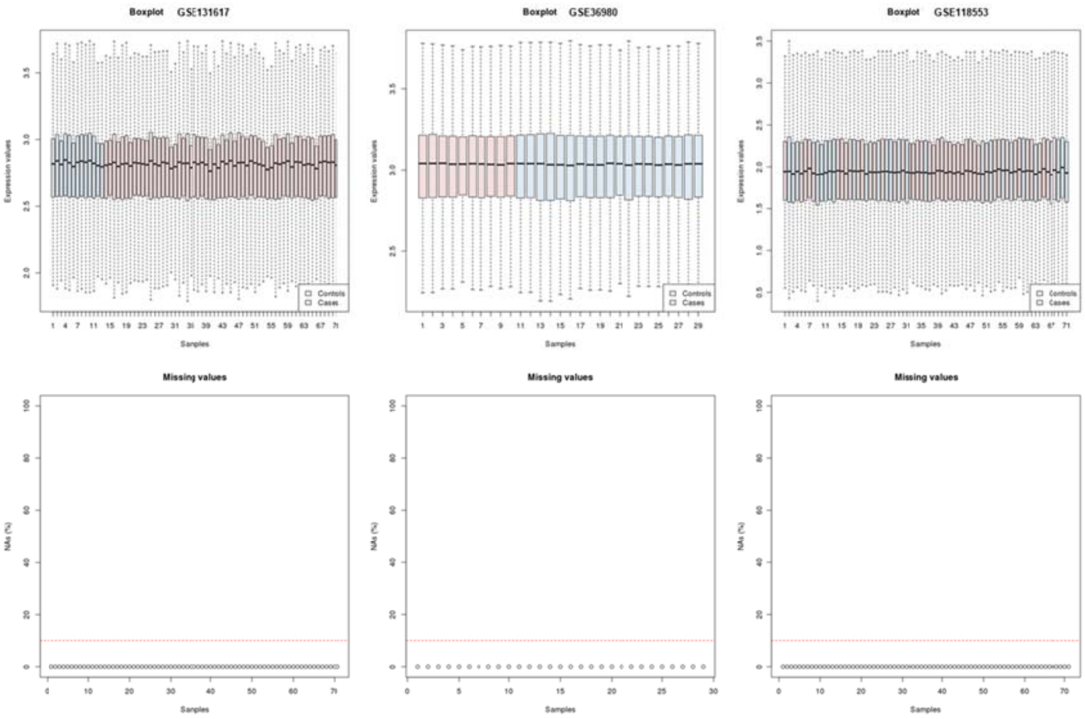

Figure S5. Heatmap of DEGs in TC datasets.

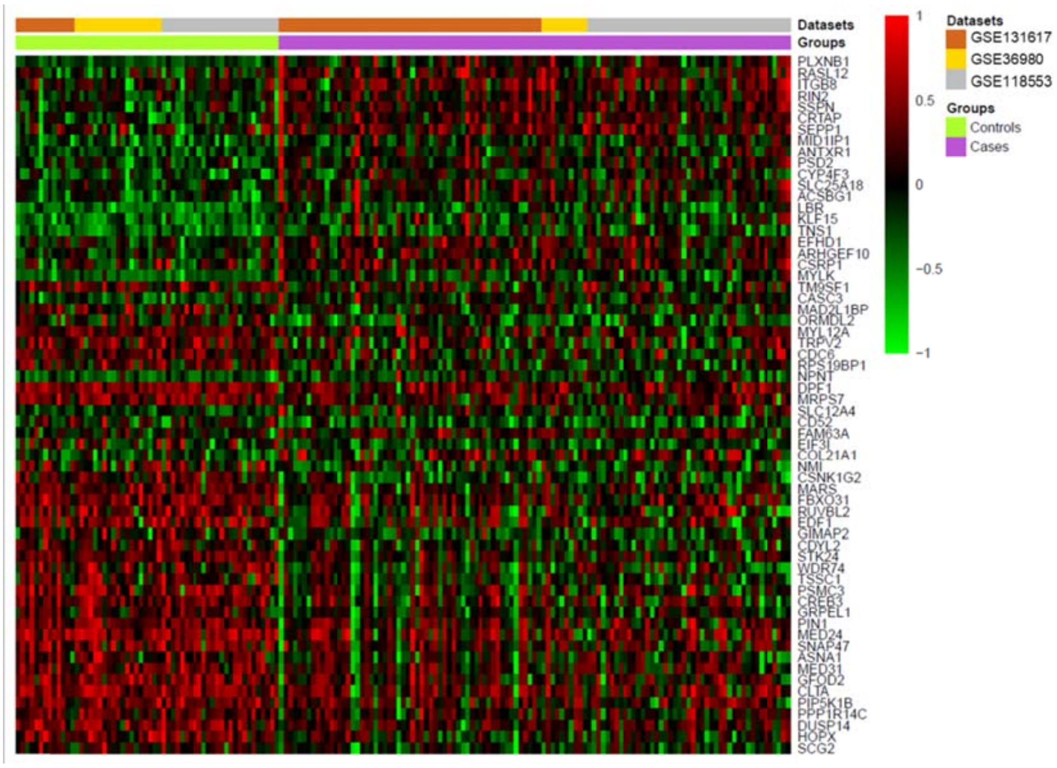

Supplement: Supplementary file 4 [file Data_Sheet_1.PDF]
